# Lung ultrasound in cardiac rehabilitation: expert consensus on protocols, clinical use, and integration into patient management from the working group on cardiac rehabilitation and cardiovascular prevention of the Italian society of cardiology

**DOI:** 10.1007/s10741-025-10560-9

**Published:** 2025-09-22

**Authors:** Costantino Mancusi, Francesco Giallauria, Mara Piccoli, Valeria Visco, Arturo Cesaro, Gerardina Fratianni, Elio Venturini, Nidal Turkmani, Antonello D’Andrea, Mario Pacileo, Raffaele Carluccio, Alessandro Maloberti, Nicola De Luca, Stefania Paolillo, Savina Nodari, Mauro Maniscalco, Pasquale Ambrosino, Alberto Palazzuoli, Paolo Calabrò, Michele Ciccarelli

**Affiliations:** 1 Department of Advanced Biomedical Sciences, University of Naples “Federico II”, Naples, Italy; 2 Department of Translational Medical Sciences, University of Naples “Federico II”, Naples, Italy; 3 Cardiology Department, “Giovan Battista Grassi” Hospital, Rome, Italy; 4 Department of Medicine, Surgery and Dentistry, University of Salerno, Fisciano, Italy; 5 Division of Clinical Cardiology, AORN “Sant’Anna e San Sebastiano”, Caserta and Department of Translational Medical Sciences, University of Campania “Luigi Vanvitelli”, Naples, Italy; 6 Department of Cardiology, Cardiac Rehabilitation Unit, IRCCS, Istituto Auxologico Italiano, “San Luca” Hospital, Milan, Italy; 7 Department of Cardiac Rehabilitation, Cecina Civil Hospital, Cecina, Italy; 8 Rehabilitation Clinic “Mons. Giosuè Calaciura”, Biancavilla and Cardiovascular Diagnostic Center, Catania, Italy; 9 Department of Cardiology and Intensive Coronary Care, “Umberto I” Hospital, Nocera Inferiore, Italy; 10 S.C. Cardiologia 4 Diagnostica e Riabilitativa - Ospedale Niguarda, Milan, Italy; 11 Scuola di Medicina e Chirurgia - Università Milano-Bicocca, Bergamo, Italy; 12 Department of Cardiology, University of Brescia and ASST “Ospedali Civili” Hospital, Brescia, Italy; 13 Istituti Clinici Scientifici Maugeri IRCCS, Pulmonary Rehabilitation Unit of Telese Terme Institute, Telese Terme, Italy; 14 Department of Clinical Medicine and Surgery, Federico II University, Naples, Italy; 15 Istituti Clinici Scientifici Maugeri IRCCS, Scientific Directorate of Telese Terme Institute, Telese Terme, Italy; 16 Cardio-Thoracic and Vascular Department, “Santa Maria alle Scotte” Hospital, University of Siena, Siena, Italy

**Keywords:** Lung, Ultrasound, Echocardiography, Cardiac, Rehabilitation, Prevention

## Abstract

Lung ultrasonography (LUS) is a reliable and reproducible tool across various clinical settings. Its high diagnostic accuracy, portability, and real-time imaging capabilities make it especially suitable for use in Emergency Departments, Intensive Care Units, and outpatient clinics. LUS has proven particularly effective in evaluating lung congestion. LUS also provides superior diagnostic accuracy for detecting pleural effusion and lung consolidations, offering real-time imaging with high spatial resolution and enabling precise monitoring throughout hospitalization. In the Cardiac Rehabilitation Unit, the routine use of LUS represents a reliable imaging modality for assessing patients with complex clinical conditions. In fact, early identification of lung congestion, pleural effusion, or lung consolidation in patients recovering from acute coronary syndrome, acute heart failure, or cardiac surgery is crucial for optimizing clinical management. Moreover, continuous monitoring of lung congestion can aid in the appropriate adjustment of diuretic therapy and exercise intensity. This review aims to present the latest evidence and recommendations for the use of LUS in the cardiac rehabilitation setting.

## Introduction

Over the last 20 years, scientific interest in lung ultrasonography (LUS) has increased exponentially. Compared to other imaging modalities, such as chest x-ray (CXR) and computed tomography (CT), LUS offers numerous advantages, including the use of non-ionizing radiation, reduced equipment costs, real-time imaging, portability, and bedside availability [1]. As a result, the adoption of LUS for diagnostic imaging of the lung and pleural space has grown significantly since 2012, and the spread of coronavirus disease 2019 (COVID-19) has accelerated the need for a standardized, evidence-based approach in the international community [2]. LUS provides a highly versatile and valuable diagnostic tool in many conditions that cardiologists encounter every day in their clinical practice. In particular, LUS has been shown to be additive to echocardiography and superior to auscultation and CXR in identifying acute decompensated heart failure (ADHF), providing rapid and accurate support in the diagnostic process of dyspnea, respiratory failure, and shock [3–5]. Therefore, the use of LUS has recently been expanded to include the assessment of cardiac and pulmonary conditions commonly seen in Cardiac Rehabilitation (CR) units, especially in patients recovering from post-cardiac surgery and acute coronary syndrome [6].

Despite that current recommendations for the management of patients admitted in CR programs do not provide specific indications for the routine use of LUS, limiting its role for the assessment of congestion in patients with HF.

This Expert Opinion of the Working Group on Cardiac Rehabilitation and Cardiovascular Prevention of the Italian Society of Cardiology provides a global overview of the usefulness of LUS in CR through a comprehensive literature review. We provide updated evidence regarding scanning protocols, indications, and clinical applications of LUS, highlighting its role as an essential tool for managing the rehabilitation process.

## How? Scanning protocols and pathological findings

LUS can be performed using different ultrasound machines, depending on the clinical setting and availability. Phased-array (“cardiac”), convex (“abdominal”), and linear (“vascular”) probes can be useful for LUS. In particular, the convex probe, due to its intermediate frequency range, provides a balanced visualization of the pleural line and subpleural space while maintaining a comprehensive view of the chest. The linear probe may be particularly useful for evaluating the pleural line, especially when pneumothorax (PNX) is suspected. The phased-array probe can be used to integrate LUS with echocardiography. However, due to its reduced spatial resolution and limited bandwidth, it is not routinely recommended. For the assessment of pleural effusion and B-lines, any type of transducer can be utilized, ensuring that the absence of an “ideal” probe does not prevent scanning a patient when clinically indicated. According to current guidelines, the convex probe (3–7 MHz) should be ideally used to perform a full lung examination. Based on the clinical setting, different ultrasound machines can be used for LUS examinations. Various studies have demonstrated that standard, portable, and handheld ultrasound devices provide adequate accuracy in evaluating major lung diseases [7–9]. The “abdominal” preset for the convex probe and the “cardiac” preset for the phased-array probe typically provide adequate image quality. For assessing the pleural line, the “superficial” preset on the linear probe is preferable. Additionally, some ultrasound machines offer a dedicated “lung” preset. Various protocols have been proposed for LUS examinations, depending on the clinical setting.

An important indication is to perform LUS on the possibly largest area of the chest available during the examination, with limitations justified by the patient’s clinical condition, including their ability to cooperate and any chest conditions such as scars or obesity. The probe should be positioned in the intercostal space with an orientation parallel to the ribs. The image depth depends on the size of the patient but is usually set to ∼10–12 cm. Gain and focus settings should be optimized for pleural line visualization by keeping the probe in the same position for at least one breath cycle. The footage should be recorded in each scan zone with a suggested duration of approximately 4–6 s. In each area of the chest, the operator must scan the entire accessible chest surface to increase sensitivity. The patient should be kept in a sitting or semi-orthopnoea position, starting the examination on each hemithorax. Intercostal scans should be performed to better examine the peripheral lung parenchyma by focusing and highlighting subpleural findings and avoiding shadowing from the ribs.

Different protocols have been tested using between 2 and 14 scanning zones per hemithorax, demonstrating the ability to accurately assess lung congestion in both acute and chronic heart failure (HF) settings [9–12]. Even for detecting lung consolidation, a 6-zone scanning protocol has been shown to be accurate [13]. In studies assessing the degree of fluid overload in dialysis patients, 28 scan protocols (16 scans from the right half and 12 scans from the left anterolateral surface of the chest) have been reported [14].

In the context of a CR unit, a complete and standardized protocol should be employed for the assessment of lung congestion, pleural effusion, and lung consolidation. We recommend adopting a standardized LUS protocol for all patients upon admission to the Cardiac Rehabilitation Unit. Lung ultrasound should be performed with 6 scans on each hemithorax (anterior, lateral, and posterior scanning zones, divided by the anterior and posterior axillary lines and upper and lower regions), covering 12 imaging areas, in all patients in the supine position at rest. For the posterior scan, the patient can sit, as long as there are no contraindications, which allows for better visualization of the posterior recesses. For each scan, the specific physiological pattern or any pathological findings should be documented, including:

- **“A”:** A-lines.
- **“B”:** B-lines.
- **“W”:** Confluent B-lines.
- **“C”:** Consolidation.
- **“PNX”:** Pneumothorax.
- **“PE”:** Pleural effusion.

**Fig. 1:**
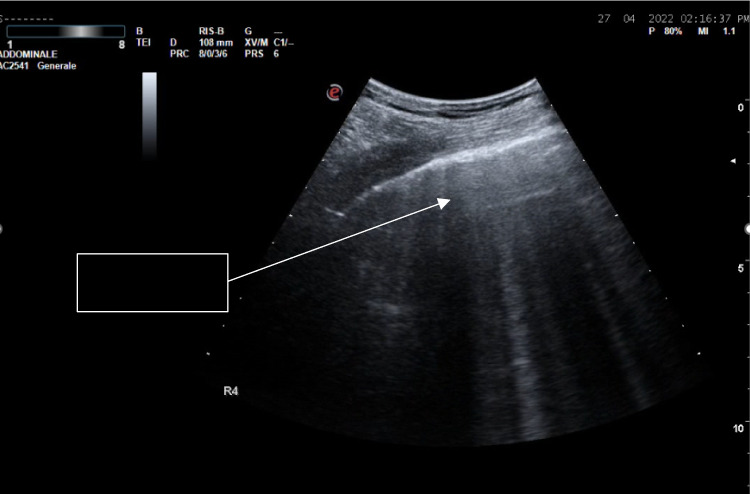
Lung Ultrasound (LUS) finding: B-lines in a patient with acute interstitial syndrome

We strongly recommend the use of the Lung Ultrasound Score (LUS Score) for monitoring lung aeration, as it has demonstrated strong prognostic accuracy [11]. A LUS score for lung aeration can be calculated for each region of interest, with points allocated according to the worst ultrasound pattern observed:

- **“0” (pattern 0):** Presence of A-lines beyond the pleural line (normal pulmonary aeration).
- **“1” (pattern 1):** Presence of multiple, well-separated vertical B-lines (more than 3) (moderate decrease in lung aeration due to interstitial syndrome) (Fig. 1).
- **“2” (pattern 2):** Presence of confluent B-lines affecting less than 50% of the image (severe decrease in lung aeration due to pulmonary oedema or confluent bronchopneumonia).
- **“3” (pattern 3):** Presence of confluent B-lines affecting more than 50% of the image (severe decrease in lung aeration due to pulmonary oedema or confluent bronchopneumonia).
- **“C”:** Presence of lung consolidation (complete loss of aeration, with persistent aeration of distal bronchioles as dynamic bronchograms).

**Fig. 2:**
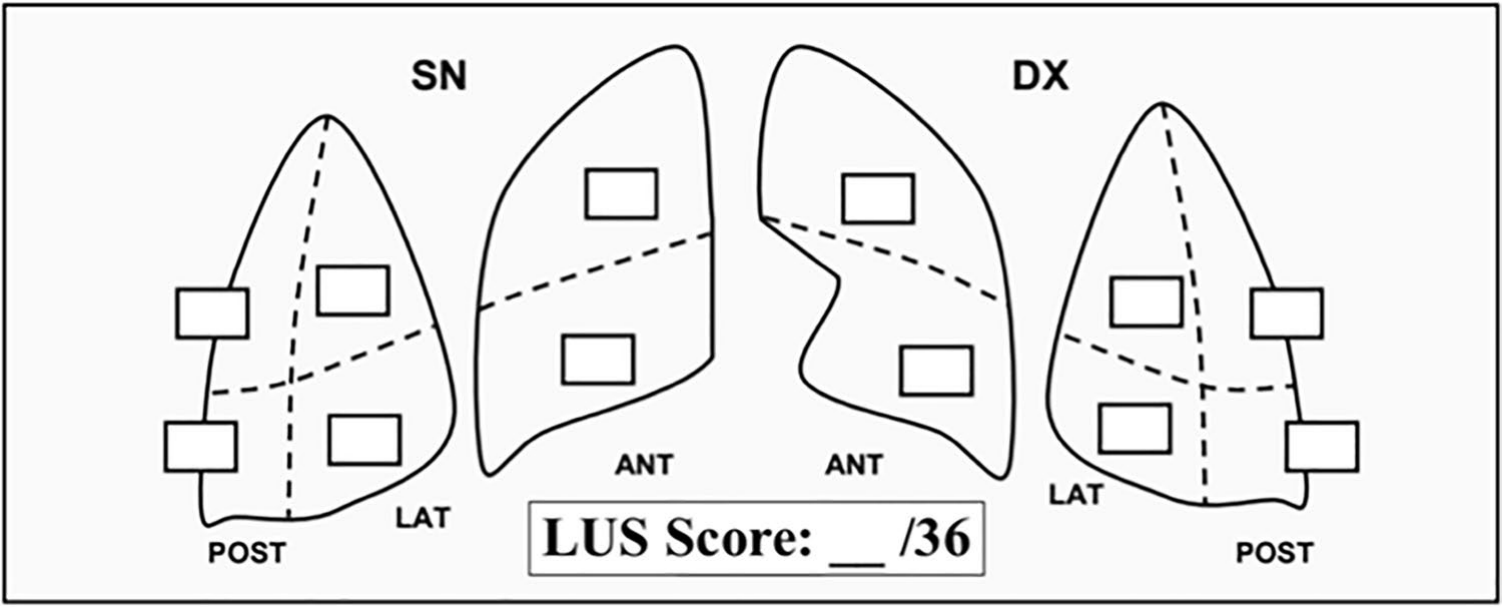
Proposal for lung ultrasound (LUS) report. 0: (pattern 0) presence of A-lines beyond the pleural line. 1: (pattern 1) presence of multiple, more than 3 and well-separated vertical B-lines. 2: (pattern 2) presence of confluent B-lines, involving less than 50% of the images. 3: (pattern 3) presence of confluent B-lines, involving more than 50% of the images. C: presence of lung consolidation: complete loss of aeration

The LUS score, ranging from 0 to 36, is the sum of each region and provides a global assessment of lung aeration, which can be regularly monitored. During cardiac rehabilitation, a reduction in the LUS score and lung congestion should be a primary goal of the rehabilitation program (Fig. 2) following the 12-zone protocol and maintaining the patient in the same position during serial examinations. Some studies have evaluated the possibility of using a count-based method for the quantification of B-lines, in which the sum of B-lines in one intercostal space per zone across all zones is reported [15]. This method is useful for the quantification of total lung congestion but does not give any topographic information on the distribution of B-lines, which is useful for the diagnostic approach. In the evaluation of patients with B-lines, it is crucial to assess echographic characteristics, which are useful to differentiate patterns of acute interstitial syndrome, including acute heart failure or acute respiratory distress syndrome (ARDS). In patients with heart failure, B-lines are usually homogeneously distributed with a typical gravity pattern and no main alteration in the pleural line. In patients with ARDS, B-lines are typically more heterogeneously distributed, especially in non-gravity-dependent regions, and are often accompanied by pleural line abnormalities and subpleural consolidations [16].

## When? Clinical settings and indications

Dyspnea on exertion is the cardinal symptom reported by patients with heart failure with both reduced (HFrEF) and preserved ejection fraction (HFpEF) and an increase in extravascular lung water (EVLW) during rest and exercise contributes to symptom occurrence [17]. LUS is a reliable and reproducible tool to assess EVLW, with B-lines homogeneously distributed with a typical gravity pattern being the hallmark. The complete mechanisms determining the development of B‐lines during exercise in HF patients are not entirely understood, evidence being scarce, mainly deriving from mixed cohorts. According to previous reports, submaximal exercise results in an increase in B‐lines in HF patients. These changes are mirrored by significant variations in natriuretic peptides, tricuspid regurgitation velocities, and left ventricular (LV) E/E′, as expressions of dynamic diastolic dysfunction [18–21]. Also, reduced LV contractile reserve seems to contribute to the development of lung congestion during exercise, even at submaximal workloads, as assessed by strain and strain rate parameters, while left ventricular ejection fraction (LVEF) is usually not sufficiently sensitive to capture this subtle systolic impairment. Interestingly, recent studies in HF and ischemic patients, using myocardial work (MW) analysis, have identified a strong correlation between LV myocardial efficiency, peak effort capacity, diastolic function, and signs of pulmonary congestion assessed by LUS during various rehabilitation training protocols [22, 23]. In addition, the appearance or increase of B‐lines during submaximal exercise has been associated with a rise in both E/E′ and with impairment in right ventricular (RV)‐to‐pulmonary circulation coupling, as assessed by respective ratios of tricuspid annular plane systolic excursion (TAPSE) or RV S′ and invasive mean pulmonary arterial pressure [24].

In various studies, the prognostic value of LUS as part of a multi‐parametric score in a mixed cohort of patients with HFpEF or at risk of developing HF has been described. In particular, multivariable models identified a change in B‐lines >10 (from rest to peak) as an independent predictor of cardiovascular death or HF hospitalization, along with peak oxygen consumption <16 mL/kg/min, minute ventilation/carbon dioxide production slope >36, pulmonary artery systolic pressure (PASP) > 50 mmHg, and resting N‐terminal pro B‐type natriuretic peptide (NT-proBNP) > 900 pg/mL. A weighted risk score (ranging from 0 to 9) including those variables accurately predicted adverse events during a median follow‐up of 18.5 months. Among those predictors, B‐lines change > 10 showed the highest association with the combined endpoint (hazard ratio: 7.81, *P* < 0.001) [25].

Overall, given that postoperative pulmonary complications (PPCs) remain a major issue after cardiac surgery (with a prevalence of at least 55%), significantly affecting morbidity, mortality, and length of hospital stay [26], LUS is an ideal tool for the rapid diagnosis, follow-up, and treatment of PPCs, complementing clinical evaluation. Compared to radiological investigations, including CXR and CT, LUS offers the advantages of lower cost, easy repeatability, and reduced exposure to ionizing radiation, while maintaining high diagnostic accuracy [27, 28]. Additionally, this technique can be performed not only by physicians but also by nurses and physiotherapists [28, 29]. For the latter, its usefulness in setting up and verifying the rehabilitation program is evident. The timing of LUS execution is not yet well defined and can vary during hospitalization [30–33]. LUS is rarely performed preoperatively [34], and the availability of a trained technician capable of performing it may be a limiting factor.

We strongly suggest performing LUS on admission in CR unit to quantify lung congestion and to evaluate presence of atelectasis, consolidation, pleural effusion, pneumothorax, or diaphragm dysfunction. LUS should be repeated after implementation of medical therapy and functional capacity and once again at the end of the rehabilitation program since residual congestion at discharge is associated with subsequent incident CV events [10–12].

### Atelectasis, consolidation, pneumonia

**Fig. 3:**
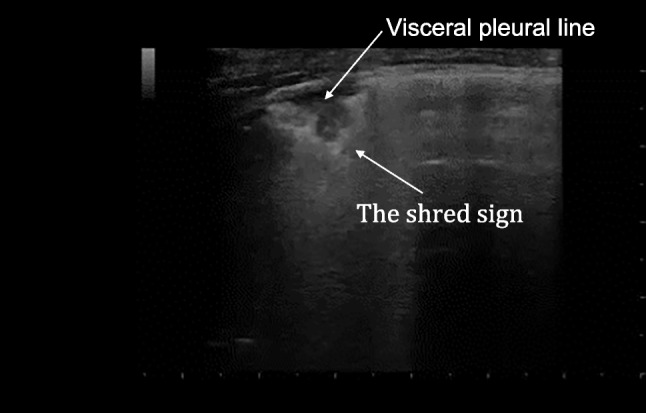
Lung ultrasound (LUS) finding: lung consolidation

Atelectasis is a common complication following all types of surgery, with variable clinical manifestations, particularly in patients who have undergone cardiac surgery. The diagnostic accuracy of LUS surpasses that of auscultation, with a sensitivity of 86% compared to 21% [35, 36]. Furthermore, LUS provides more precise anatomical-regional localization. When compared to CXR, LUS also proves superior, with its diagnostic capacity increasing from the first to the third postoperative day (LUS 98.3% *vs.* CXR 67.8%, *p* < 0.001 on day 3) [37, 38]. In the context of consolidations, which can result from various causes (*e.g.*, embolization, contusion, infection, or cancer), LUS can aid in the diagnosis of pneumonia based on the presence of a positive air bronchogram, abnormal pleural line, pleural effusion, in addition to the consolidation itself characterized by abnormal shred sign (Fig. 3) [34]. The clinical evaluation of the patient, combined with the presence of fever or signs of inflammation (*i.e.*, leucocytosis, increased C-reactive protein, and procalcitonin), facilitates the differential diagnosis.

### Pleural effusion

**Fig. 4:**
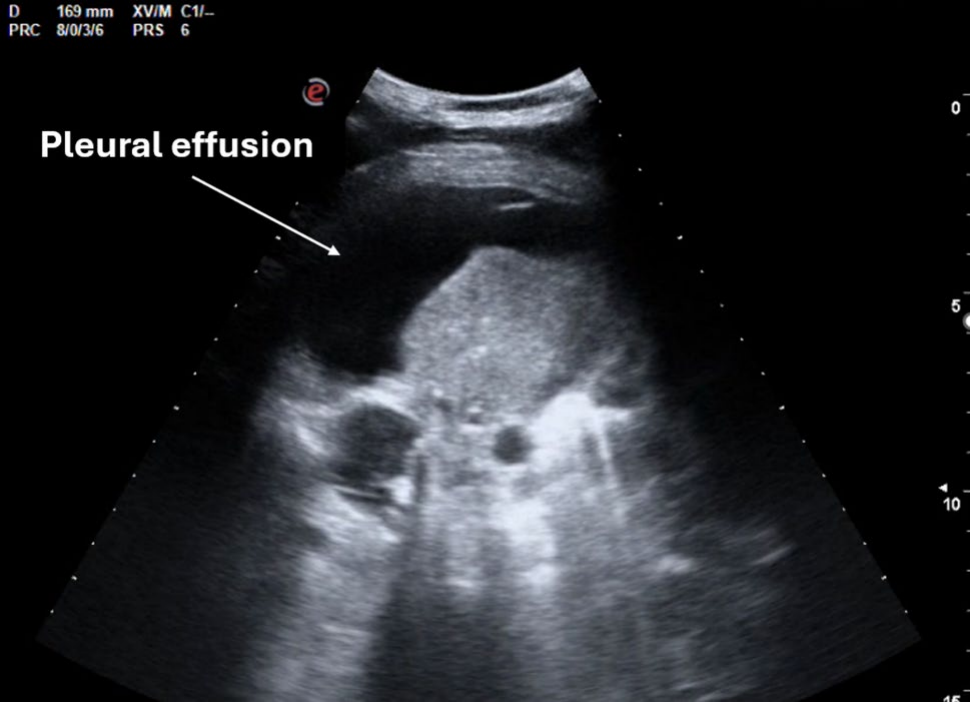
Lung ultrasound (LUS) finding:large pleural effusion

For pleural effusion evaluation, LUS offers several advantages. First, by analysing the echogenic characteristics of the fluid, it helps differentiate between transudate and exudate (Fig. 4). Additionally, it facilitates thoracentesis while minimizing complications, particularly pneumothorax. Moreover, LUS has a diagnostic sensitivity and specificity comparable to CT [35, 36], enabling accurate and individualized monitoring of pleural effusion during hospitalization. While formulas have been proposed for quantitative assessment, including in patients undergoing cardiac surgery, pleural effusion is usually classified qualitatively in clinical practice as mild, moderate, or severe based on the number of intercostal spaces involved.

### Pneumothorax

Compared to CXR, LUS has superior sensitivity while maintaining the same specificity for pneumothorax [38]. Key diagnostic findings include the absence of lung sliding and B-lines. Furthermore, when present, the “lung point” defined as the transition point where the pleura stops moving, has 100% specificity but low sensitivity [36].

### Diaphragm dysfunction

Using subxiphoid M-mode, diaphragmatic movement can be classified as normal (difference in excursion between the two hemidiaphragms <50%), reduced (>50%), absent (flat line), or paradoxical (motion away from the transducer) [27]. The severity of movement abnormality correlates with an increased risk of atelectasis. More simply, an excursion <2 cm is considered indicative of diaphragm dysfunction, a common condition affecting up to 70% of patients after cardiac surgery. Rehabilitation treatment can restore diaphragmatic motility in over 50% of patients enrolled in a Cardiac Rehabilitation program, with recovery associated with improved functional capacity, as assessed by the 6-min walking test [39].

### Lung congestion

The presence of homogeneously distributed with typical gravity pattern B-lines on LUS is indicative of increased EVLW and worsening pulmonary congestion. The detection of three or more B-lines in each scanning area indicates progressive parenchymal deaeration, with further increases reflecting greater EVLW accumulation and worsening congestion. Therefore, a correlation has been observed between the number of B-lines and several clinical factors, including the duration of coronary artery bypass graft surgery, length of mechanical ventilation, risk of deoxygenation, and length of stay in the intensive care unit [30]. Given its superior diagnostic accuracy for detecting pulmonary oedema compared to CXR [32], LUS can effectively guide treatment decisions, including the use of inotropes and/or diuretics, to optimize fluid balance.

## Why? Rationale and implications

Chronic HF is characterized by neurohumoral activation and sodium retention, leading to excessive fluid accumulation in both the systemic and pulmonary circulations [40, 41]. Several registries (*e.g.*, ADHERE, OPTIMIZE-HF, and the EuroHeart Failure survey) have shown that acute HF hospitalizations are primarily driven by volume overload rather than low cardiac output in most cases [42]. Analogous to fluid mechanics, if the pulmonary vascular bed is considered a confined system, congestion can be described as an increase in the weight of the fluid column. Since force is transmitted through a fluid as a pressure wave, pulmonary capillary wedge pressure (PCWP) serves as a reasonable estimate of the pressure across this fluid column. An elevated PCWP indicates a state of “hemodynamic congestion”. As a result, an increased PCWP can cause the redistribution of excess fluid within the lungs, leading to interstitial and alveolar oedema, collectively referred to as PC, which manifests clinically as dyspnoea, orthopnoea, pulmonary rales, peripheral oedema, and jugular venous distension [42–45]. Although the development of these congestive signs and symptoms represents the main reason for hospitalization in HF patients, this often occurs several days after the onset of PCWP elevation [46–50]. However, the distribution of excess fluid within the lungs depends on hydrostatic pressure and several other factors, including plasma oncotic pressure, permeability and integrity of the alveolar-capillary membrane, and lymphatic drainage. Accordingly, some HF patients can tolerate even marked LA pressure elevations without developing clinical or radiographic lung congestion [51]. Indeed, the extent of lung congestion has been linked not only to markers of capillary hydrostatic pressure (PAWP and NT-proBNP) and plasma oncotic pressure (hypoalbuminemia) [41] but also to anaemia, elevated pulmonary vascular resistance, declining renal function, and the presence of coronary artery disease (CAD) [52].

On the one hand, natriuretic peptides may directly enhance capillary permeability [53]. Moreover, as observed in chronic kidney disease, congestion worsens with declining renal function, likely due to a combination of high hydrostatic pressure from hypervolemia and direct effects on capillary permeability [54]. The strongest correlates of congestion include low haemoglobin, anaemia, iron deficiency, reduced oncotic pressure, and haemodilution due to expanded circulating volume [55]. CAD has also been linked to more severe lung congestion, possibly due to proinflammatory cytokines contributing to increased capillary permeability [56]. Additionally, the renin–angiotensin system likely plays a role, as previous studies have demonstrated that angiotensin-converting enzyme (ACE) inhibition protects against fluid extravasation from lung capillaries, thereby improving gas diffusion [57]. Furthermore, increased LV filling pressures augment LV wall stress, leading to chamber dilation and increased ventricular sphericity, which may result in mitral regurgitation, further contributing to hemodynamic deterioration and ventricular enlargement [51].

On the other hand, pulmonary adaptation to increased post-capillary pressure in HF is believed to involve reduced capillary filtration due to basal membrane thickening [58], enhanced alveolar fluid clearance [58], and increased lymphatic drainage [40, 58, 59]. While radiological scoring has been correlated with mortality, no direct relationship has been found with hemodynamic data. These findings align with the understanding that congestion results from multiple mechanisms driving HF progression and that congestion persists longer than transient hemodynamic changes [52]. Finally, patient-related factors, such as excessive salt and water intake, nonadherence to medication regimens, and the use of cardiotoxic substances (*e.g.*, alcohol, cocaine) may also contribute to congestion [52].

In the assessment of lung congestion, LUS has shown excellent diagnostic accuracy [58]. Pulmonary tissue oedema or congestion is identified by the presence of comet-tail artifacts or B-lines homogeneously distributed with a typical gravity pattern [60, 61]. The distance between B-lines at the pleura can help differentiate the localization of oedema. B-lines separated by approximately 7 mm correspond to interstitial oedema, whereas a 3 mm distance indicates the presence of alveolar oedema [62]. Ultrasound cannot distinguish the nature of the fluid (water, pus) or proliferating tissue (fibrotic, infiltrative), nor the mechanism causing the passage from the vessel to the interstitium (hydrostatic pressure or increased permeability) [63]. The differential diagnosis of conditions causing this syndrome should consider additional characteristics. A hallmark of permeability-increased oedema is the presence of patchy areas (normal lung regions adjacent to pathological ones), absence of pleuropulmonary sliding, and consolidations. Some authors have attempted to correlate the severity of alveolar-interstitial syndrome with the extent of pulmonary oedema, using EVLW measurement as a reference. Agricola et al*.* [64] proposed an ultrasonographic scoring system based on the sum of B-lines homogeneously distributed with a typical gravity pattern observed in scanned areas, which has shown moderate correlation with EVLW measured by transpulmonary thermodilution. This score serves as a useful semi-quantitative estimation of pulmonary oedema in clinical practice. Based on the score obtained, oedema can be classified as absent (≤ 5 B-lines), mild (5–15 B-lines), moderate (15–30 B-lines), or severe (more than 30 B-lines) [64].

Similarly, LUS offers superior diagnostic performance in detecting pleural effusion. Moreover, it enables dynamic assessment, facilitating the evaluation of pleural fluid movement with changes in patient position or respiratory effort [63, 65]. Confirming the diagnosis is the first step in assessing patients suspected of having pleural effusion, particularly when a white hemithorax is observed on CXR [66]. Ultrasound is a valuable tool in this context, allowing differentiation between effusion and lung consolidations while demonstrating superior accuracy compared to bedside CXR (93% *vs.* 47%) [66]. Notably, CXR is limited in detecting pleural effusion, particularly in patients in the orthostatic position, and is generally effective only when effusion volumes exceed 200 mL. In contrast, ultrasound can detect effusions as small as 20 mL, regardless of patient positioning [67, 68].

**Fig. 5:**
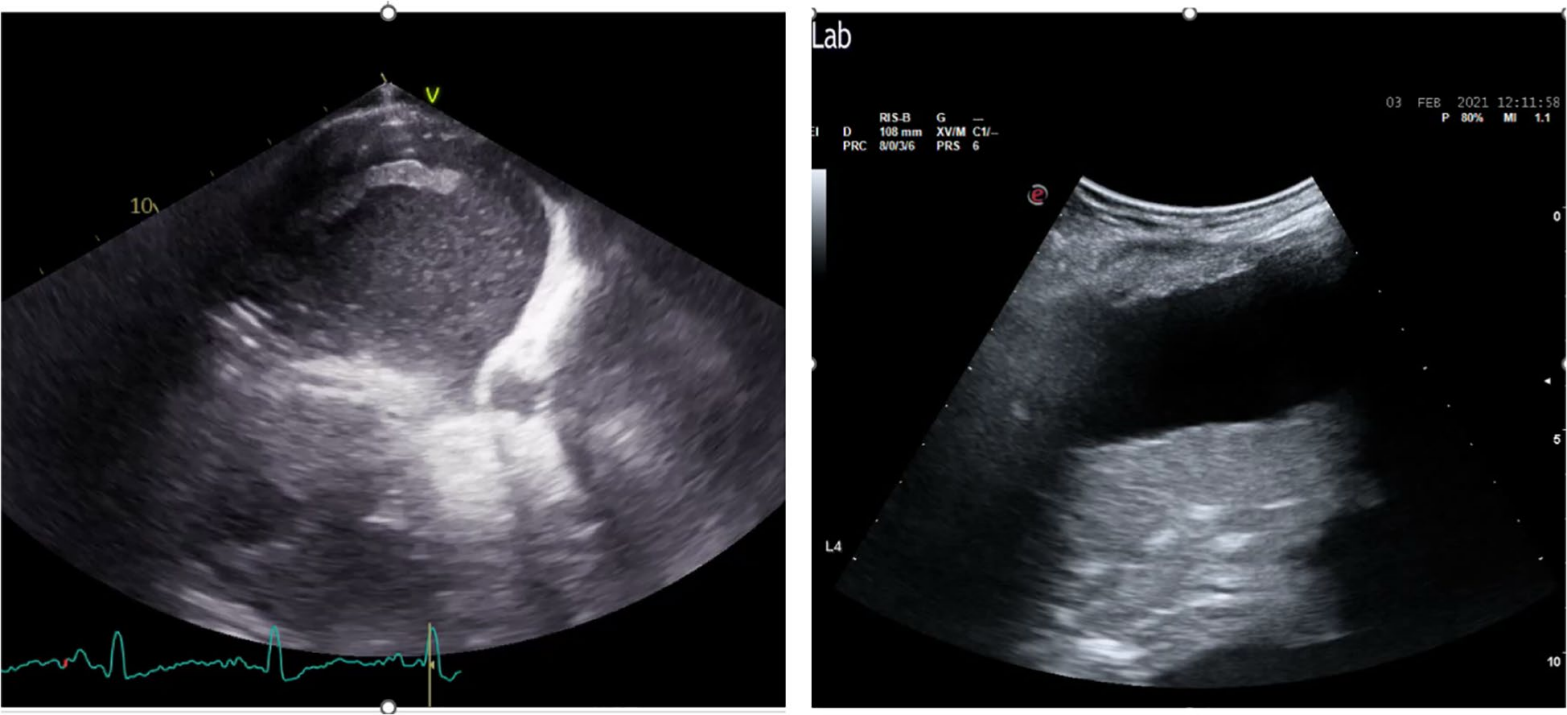
Lung Ultrasound (LUS) finding: pleural effusion characterized by exudate on the left panel and transudate on the right panel

The next step involves distinguishing between transudative and exudative pleural effusions. The ultrasound characteristics of the effusion can provide initial diagnostic clues, with appearances ranging from anechoic to complex non-septate, complex septate, or homogeneously echogenic (Fig. 5). However, a definitive diagnosis requires thoracentesis for comprehensive physical, chemical, and microbiological analysis of the fluid. Typically, complex effusions suggest an exudative process, while anechoic effusions are more likely to be transudative. Nonetheless, exceptions exist, as some transudative effusions may present as complex non-septate due to the presence of cells, proteins, and lipids. Conversely, some exudative effusions may appear anechoic. Homogeneous echogenic effusions generally indicate haemorrhagic effusions or empyema [69].

Several studies have assessed the diagnostic accuracy of LUS for lung congestion and pleural effusion across different patient populations and clinical settings. Meta-analyses have consistently demonstrated high sensitivity and specificity for detecting these pulmonary abnormalities, with pooled estimates exceeding 90% for both parameters [70]. Furthermore, LUS has shown excellent agreement with reference standards such as CXR and CT, thus reinforcing its role as a reliable diagnostic tool for respiratory pathology [71].

In hospitalized HF patients, the number of B-lines detected by LUS at admission [10, 72] or discharge [73–75] has been associated with subsequent outcomes. Studies have used different outcome definitions, with some considering only HF hospitalization [72, 75] and others including a composite measure that also accounts for all-cause mortality [10, 74]. Follow-up durations have varied, ranging from 3 [72] to 6 [10, 73, 75] or 12 months [76], but results have remained consistent across studies. However, there is no clear consensus on the optimal B-line threshold, which has ranged from 5 [73] to 22 [74]. These associations have been observed regardless of EF category (reduced [76, 77], mildly reduced [10, 75, 78], or preserved [76]) and have also been confirmed in chronic HF outpatients [76, 78].

Only two trials have assessed the prognostic impact of LUS-guided decongestion therapies. In the first, 20 patients managed with LUS guidance (compared to one hundred receiving standard care) experienced greater decongestion over a shorter hospital stay, along with a significant reduction in adverse outcomes [77]. In the second study, patients recently hospitalized for HF were randomized to either LUS-guided management or standard care during outpatient follow-up (61 *vs.* 62 patients). Those in the LUS-guided group had a significant reduction in subsequent HF hospitalizations, all-cause mortality, or urgent visits for HF symptoms, with the latter being the primary driver of the composite outcome [76].

In patients with severe aortic stenosis (AS), HF and pulmonary congestion (PC) are common and nearly universal pathophysiological features [63, 79–81]. LUS assessment of B-lines in AS reliably reflects structural and functional impairment associated with severe valvular heart disease, as well as the hemodynamic consequences of AS. This technique provides insights into a distinct pathophysiological target (the alveolar-capillary barrier rather than the physiologically critical epicardial artery stenosis), identifies a different clinical marker (B-lines instead of regional wall motion abnormalities), and captures these changes in a different temporal phase (post-stress rather than at peak stress) [3]. The presence of severe B-lines (≥30) strongly predicts adverse events. Current guidelines recommend valve replacement when a comprehensive assessment (*i.e.*, pressure gradients, aortic valve area, valve calcification severity, and flow parameters) confirms severe AS, accompanied by signs of LV decompensation detected through echocardiographic measurements or the onset of symptoms [79, 82]. Stress echocardiography and cardiac magnetic resonance (CMR), both strong predictors for AS, are not always available, and repeated measurements are not feasible [79]. Integrating LUS with standard transthoracic echocardiography (TTE) or incorporating LUS into stress echocardiography protocols could enhance risk stratification. Cardiac damage, involving LV, mitral valve, and left atrium (LA), leads to PC, contributing to HF signs and symptoms. Therefore, early detection of PC via LUS may offer additional prognostic and diagnostic value. A more precise evaluation of PC may serve as a promising tool for identifying high-risk patients, optimizing the timing of interventional procedures such as transcatheter aortic valve replacement (TAVR) or open-heart surgery, and refining HF management strategies [79]. Furthermore, LUS may impact postoperative outcomes; however, further studies are required to validate these hypotheses. B-lines are valuable in differentiating acute HF syndromes from noncardiac causes of acute dyspnea in emergency settings, demonstrating high sensitivity and specificity [79, 83, 84].

## Conclusions

The present review underscores the importance of LUS as a fundamental tool for evaluating patients referred to Cardiac Rehabilitation programs. Considering patients’ comorbidities and the need to monitor lung congestion, LUS provides a unique diagnostic opportunity to tailor both therapy and exercise. Multi-centre studies are needed to define its role throughout the entire rehabilitation process and to assess the beneficial effect of early identification and management of clinical conditions such as atelectasis, consolidation, pneumonia, pleural effusion, pneumothorax, diaphragm dysfunction, and lung congestion. The potential role of artificial intelligence in this setting is emerging to guide the acquisition of diagnostic-quality LUS images by non-expert health care professionals and to provide specific tools for the advanced imaging analysis able to distinguish different patterns of acute interstitial syndrome.

## Data Availability

No datasets were generated or analysed during the current study.

## References

[1] Inchingolo R, Zanforlin A, Buonsenso D et al (2024) Lung ultrasound signs: the beginning. Part 3-an Accademia di Ecografia Toracica comprehensive review on ultrasonographic signs and real needs. J Ultrasound Med 43(4):629–641 38168739 10.1002/jum.16397

[2] Demi L, Wolfram F, Klersy C et al (2023) New international guidelines and consensus on the use of lung ultrasound. J Ultrasound Med 42(2):309–344 35993596 10.1002/jum.16088 PMC10086956

[3] Picano E, Scali MC, Ciampi Q, Lichtenstein D (2018) Lung ultrasound for the cardiologist. JACC Cardiovasc Imaging 11(11):1692–1705 30409330 10.1016/j.jcmg.2018.06.023

[4] Sforza A, Carlino MV, Guarino M et al (2019) Anterior vs lateral symmetric interstitial syndrome in the diagnosis of acute heart failure. Int J Cardiol 280:130–132 30665807 10.1016/j.ijcard.2019.01.013

[5] Sforza A, Mancusi C, Carlino MV et al (2017) Diagnostic performance of multi-organ ultrasound with pocket-sized device in the management of acute dyspnea. Cardiovasc Ultrasound 15(1):16 28629375 10.1186/s12947-017-0105-8 PMC5477143

[6] Bertolone DT, De Colle C, Rozza F et al (2021) Lung ultrasound: a narrative review and proposed protocol for patients admitted to Cardiac Rehabilitation Unit. Monaldi Arch Chest Dis 92(1). 10.4081/monaldi.2021.1753 34461698

[7] Katende A, Oehri J, Urio VZ et al (2024) Use of a handheld ultrasonographic device to identify heart failure and pulmonary disease in rural Africa. JAMA Netw Open 7(2):e240577 38416495 10.1001/jamanetworkopen.2024.0577 PMC10902720

[8] Pang PS, Russell FM, Ehrman R et al (2021) Lung Ultrasound–Guided Emergency Department Management of Acute Heart Failure (BLUSHED-AHF). JACC: Heart Failure 9(9):638–648 34246609 10.1016/j.jchf.2021.05.008 PMC8419011

[9] Platz E, Lewis EF, Uno H et al (2016) Detection and prognostic value of pulmonary congestion by lung ultrasound in ambulatory heart failure patients. Eur Heart J 37(15):1244–1251 26819225 10.1093/eurheartj/ehv745 PMC5006102

[10] Platz E, Campbell RT, Claggett B et al (2019) Lung ultrasound in acute heart failure: prevalence of pulmonary congestion and short- and long-term outcomes. JACC Heart Fail 7(10):849–858 31582107 10.1016/j.jchf.2019.07.008 PMC8409324

[11] Xu P, Ye L, Li L, Huang W, Liu W, Huang K (2023) Comparison of the prognostic value, feasibility, and reproducibility among different scoring methods of 8 point lung ultrasonography in patients with acute heart failure. Intern Emerg Med 18(8):2321–2332 37747589 10.1007/s11739-023-03433-2

[12] Perillo A, Basile C, Fucile I, Rozza F, De Luca N, Mancusi C (2024) Lung ultrasound at discharge predicts outcomes in heart failure: a pilot study. J Cardiovasc Med (Hagerstown) 25(5):394–396 38526933 10.2459/JCM.0000000000001613 PMC10990019

[13] Mancusi C, Fucile I, Gargiulo P et al (2022) Lung ultrasound in coronary care unit, an important diagnostic tool for concomitant pneumonia. Diagnostics 12(12):3082 36553089 10.3390/diagnostics12123082 PMC9776793

[14] Torino C, Gargani L, Sicari R et al (2016) The agreement between auscultation and lung ultrasound in hemodialysis patients: the LUST study. Clin J Am Soc Nephrol 11(11):2005–2011 27660305 10.2215/CJN.03890416 PMC5108194

[15] Dwyer KH, Merz AA, Lewis EF et al (2018) Pulmonary congestion by lung ultrasound in ambulatory patients with heart failure with reduced or preserved ejection fraction and hypertension. J Card Fail 24(4):219–226 29499322 10.1016/j.cardfail.2018.02.004 PMC5895087

[16] Bosso G, Allegorico E, Pagano A et al (2021) Lung ultrasound as diagnostic tool for SARS-CoV-2 infection. Intern Emerg Med 16(2):471–476 33011929 10.1007/s11739-020-02512-y PMC7532928

[17] Arnone MI, Sforza A, Carlino MV et al (2023) Assessment of E/A ratio helps emergency clinicians in the management of patients with acute dyspnea. Intern Emerg Med 18(6):1823–1830 37103762 10.1007/s11739-023-03279-8 PMC10504390

[18] Simonovic D, Coiro S, Carluccio E et al (2018) Exercise elicits dynamic changes in extravascular lung water and haemodynamic congestion in heart failure patients with preserved ejection fraction. Eur J Heart Fail 20(9):1366–1369 29943885 10.1002/ejhf.1228

[19] Scali MC, Zagatina A, Ciampi Q, Stress Echo 2020 Study Group of the Italian Society of Echocardiography and Cardiovascular Imaging et al (2020) Lung ultrasound and pulmonary congestion during stress echocardiography. JACC Cardiovasc Imaging 13(10):2085–2095 32682714 10.1016/j.jcmg.2020.04.020

[20] Scali MC, Cortigiani L, Simionuc A, Gregori D, Marzilli M, Picano E (2017) Exercise-induced B-lines identify worse functional and prognostic stage in heart failure patients with depressed left ventricular ejection fraction. Eur J Heart Fail 19(11):1468–1478 28198075 10.1002/ejhf.776

[21] Agricola E, Picano E, Oppizzi M et al (2006) Assessment of stress-induced pulmonary interstitial edema by chest ultrasound during exercise echocardiography and its correlation with left ventricular function. J Am Soc Echocardiogr 19(4):457–463 16581487 10.1016/j.echo.2005.11.013

[22] D’Andrea A, Carbone A, Ilardi F et al (2022) Effects of high intensity interval training rehabilitation protocol after an acute coronary syndrome on myocardial work and atrial strain. Medicina (Kaunas) 58(3):453 35334629 10.3390/medicina58030453 PMC8955977

[23] D’Andrea A, Ilardi F, D’Ascenzi F et al (2021) Working Group of Echocardiography of the Italian Society of Cardiology (SIC). Impaired myocardial work efficiency in heart failure with preserved ejection fraction. Eur Heart J Cardiovasc Imaging 22(11):1312–1320. 10.1093/ehjci/jeab153 34410362

[24] Reddy YNV, Obokata M, Wiley B et al (2019) The haemodynamic basis of lung congestion during exercise in heart failure with preserved ejection fraction. Eur Heart J 40(45):3721–3730 31609443 10.1093/eurheartj/ehz713 PMC7963140

[25] Pugliese NR, De Biase N, Gargani L et al (2021) Predicting the transition to and progression of heart failure with preserved ejection fraction: a weighted risk score using bio-humoural, cardiopulmonary, and echocardiographic stress testing. Eur J Prev Cardiol 28(15):1650–1661 33624088 10.1093/eurjpc/zwaa129

[26] Fischer MO, Brotons F, Briant AR, VENICE study group et al (2022) Postoperative pulmonary complications after cardiac surgery: the VENICE international cohort study. J Cardiothorac Vasc Anesth 36(8 Pt A):2344–2351 35094928 10.1053/j.jvca.2021.12.024

[27] Cantinotti M, Marchese P, Giordano R et al (2022) Overview of lung ultrasound in pediatric cardiology. Diagnostics 12(3):763 35328316 10.3390/diagnostics12030763 PMC8946933

[28] Churchill LJ, Tronstad O, Mandrusiak AM et al (2024) The role of lung ultrasound for detecting atelectasis, consolidation, and/or pneumonia in the adult cardiac surgery population: a scoping review of the literature. Aust Crit Care 37(1):193–201 37709655 10.1016/j.aucc.2023.08.002

[29] Vitale J, Mumoli N, Giorgi-Pierfranceschi M et al (2016) Comparison of the accuracy of nurse-performed and physician-performed lung ultrasound in the diagnosis of cardiogenic dyspnea. Chest 150(2):470–471 27502985 10.1016/j.chest.2016.04.033

[30] Emperador F 4th, Bennett SR, Gonzalez J, Saati A, Alsaywid BS, Fernandez JA (2020) Extravascular lung water and effect on oxygenation assessed by lung ultrasound in adult cardiac surgery. Cureus 12(8):e9953 32983659 10.7759/cureus.9953 PMC7510178

[31] Touw HR, Parlevliet KL, Beerepoot M et al (2018) Lung ultrasound compared with chest X-ray in diagnosing postoperative pulmonary complications following cardiothoracic surgery: a prospective observational study. Anaesthesia 73(8):946–954 29529332 10.1111/anae.14243 PMC6099367

[32] Alsaddique A, Royse AG, Royse CF et al (2016) Repeated monitoring with transthoracic echocardiography and lung ultrasound after cardiac surgery: feasibility and impact on diagnosis. J Cardiothorac Vasc Anesth 30(2):406–412 26723882 10.1053/j.jvca.2015.08.033

[33] Cantinotti M, Giordano R, Scalese M et al (2020) Prognostic value of a new lung ultrasound score to predict intensive care unit stay in pediatric cardiac surgery. Ann Thorac Surg 109(1):178–184 31400328 10.1016/j.athoracsur.2019.06.057

[34] Song IK, Kim EH, Lee JH, Kang P, Kim HS, Kim JT (2018) Utility of perioperative lung ultrasound in pediatric cardiac surgery: a randomized controlled trial. Anesthesiology 128(4):718–727 29309282 10.1097/ALN.0000000000002069

[35] Yan JH, Yu N, Wang YH, Gao YB, Pan L (2020) Lung ultrasound vs chest radiography in the diagnosis of children pneumonia: systematic evidence. Medicine (Baltimore) 99(50):e23671 33327356 10.1097/MD.0000000000023671 PMC7738074

[36] Cantinotti M, Giordano R, Volpicelli G et al (2016) Lung ultrasound in adult and paediatric cardiac surgery: is it time for routine use? Interact Cardiovasc Thorac Surg 22(2):208–215 26586677 10.1093/icvts/ivv315

[37] Usta E, Mustafi M, Ziemer G (2010) Ultrasound estimation of volume of postoperative pleural effusion in cardiac surgery patients. Interact Cardiovasc Thorac Surg 10(2):204–207 19903687 10.1510/icvts.2009.222273

[38] Volpicelli G, Boero E, Sverzellati N et al (2014) Semi-quantification of pneumothorax volume by lung ultrasound. Intensive Care Med 40(10):1460–1467 25056671 10.1007/s00134-014-3402-9

[39] Maranta F, Cianfanelli L, Rizza V et al (2022) Diaphragm dysfunction after cardiac surgery: insights from ultrasound imaging during cardiac rehabilitation. Ultrasound Med Biol 48(7):1179–1189 35351317 10.1016/j.ultrasmedbio.2022.02.011

[40] Gheorghiade M, Follath F, Ponikowski P, European Society of Cardiology, European Society of Intensive Care Medicine et al (2010) Assessing and grading congestion in acute heart failure: a scientific statement from the acute heart failure committee of the heart failure association of the European Society of Cardiology and endorsed by the European Society of Intensive Care Medicine. Eur J Heart Fail 12(5):423–433 20354029 10.1093/eurjhf/hfq045

[41] Clark AL, Cleland JG (2013) Causes and treatment of oedema in patients with heart failure. Nat Rev Cardiol 10(3):156–170 23319101 10.1038/nrcardio.2012.191

[42] Gheorghiade M, Filippatos G, De Luca L, Burnett J (2006) Congestion in acute heart failure syndromes: an essential target of evaluation and treatment. Am J Med 119(12 Suppl 1):S3–S10 17113398 10.1016/j.amjmed.2006.09.011

[43] Gheorghiade M, Zannad F, Sopko G et al (2005) International Working Group on Acute Heart Failure Syndromes. Acute heart failure syndromes: current state and framework for future research. Circulation112(25):3958–3968 10.1161/CIRCULATIONAHA.105.590091 16365214

[44] Fonarow GC, ADHERE Scientific Advisory Committee (2003) The acute decompensated heart failure national registry (ADHERE): opportunities to improve care of patients hospitalized with acute decompensated heart failure. Rev Cardiovasc Med 4(Suppl 7):S21-30 14668697

[45] Gheorghiade M, Shin DD, Thomas TO, Brandimarte F, Fonarow GC, Abraham WT (2006) Congestion is an important diagnostic and therapeutic target in heart failure. Rev Cardiovasc Med 7(Suppl 1):S12-24 16955056

[46] Chaudhry SI, Wang Y, Concato J, Gill TM, Krumholz HM (2007) Patterns of weight change preceding hospitalization for heart failure. Circulation 116(14):1549–1554 17846286 10.1161/CIRCULATIONAHA.107.690768 PMC2892745

[47] Adamson PB, Magalski A, Braunschweig F et al (2003) Ongoing right ventricular hemodynamics in heart failure: clinical value of measurements derived from an implantable monitoring system. J Am Coll Cardiol 41(4):565–571 12598066 10.1016/s0735-1097(02)02896-6

[48] Visco V, Esposito C, Manzo M et al (2022) A multistep approach to deal with advanced heart failure: a case report on the positive effect of cardiac contractility modulation therapy on pulmonary pressure measured by CardioMEMS. Front Cardiovasc Med 9:874433 35445087 10.3389/fcvm.2022.874433 PMC9013826

[49] Visco V, Esposito C, Vitillo P, Vecchione C, Ciccarelli M (2020) It is easy to see, but it is better to foresee: a case report on the favourable alliance between CardioMEMS and levosimendan. Eur Heart J Case Rep 4(4):1–5 32974431 10.1093/ehjcr/ytaa205 PMC7501887

[50] Jordan F, Adams J, Farzami B, Kudzin ZH (1986) Conjugated alpha-keto acids as mechanism-based inactivators of brewer’s yeast pyruvate decarboxylase: electronic effects of substituents and detection of a long-lived intermediate. J Enzyme Inhib 1(2):139–149 3334240 10.3109/14756368609020112

[51] Ware LB, Matthay MA (2005) Clinical practice. Acute pulmonary edema. N Engl J Med 353(26):2788–2796 16382065 10.1056/NEJMcp052699

[52] Melenovsky V, Andersen MJ, Andress K, Reddy YN, Borlaug BA (2015) Lung congestion in chronic heart failure: haemodynamic, clinical, and prognostic implications. Eur J Heart Fail 17(11):1161–1171 26467180 10.1002/ejhf.417

[53] Chen W, Gassner B, Börner S et al (2012) Atrial natriuretic peptide enhances microvascular albumin permeability by the caveolae-mediated transcellular pathway. Cardiovasc Res 93(1):141–151 22025581 10.1093/cvr/cvr279 PMC3243041

[54] Crosbie WA, Snowden S, Parsons V (1972) Changes in lung capillary permeability in renal failure. Br Med J 4(5837):388–390 4564763 10.1136/bmj.4.5837.388 PMC1786644

[55] Androne AS, Katz SD, Lund L et al (2003) Hemodilution is common in patients with advanced heart failure. Circulation 107(2):226–229 12538419 10.1161/01.cir.0000052623.16194.80

[56] Milo O, Cotter G, Kaluski E et al (2003) Comparison of inflammatory and neurohormonal activation in cardiogenic pulmonary edema secondary to ischemic versus nonischemic causes. Am J Cardiol 92(2):222–226 12860231 10.1016/s0002-9149(03)00545-9

[57] Guazzi M, Agostoni P, Guazzi MD (2001) Modulation of alveolar-capillary sodium handling as a mechanism of protection of gas transfer by enalapril, and not by losartan, in chronic heart failure. J Am Coll Cardiol 37(2):398–406 11216953 10.1016/s0735-1097(00)01131-1

[58] Huang W, Kingsbury MP, Turner MA, Donnelly JL, Flores NA, Sheridan DJ (2001) Capillary filtration is reduced in lungs adapted to chronic heart failure: morphological and haemodynamic correlates. Cardiovasc Res 49(1):207–217 11121813 10.1016/s0008-6363(00)00223-6

[59] Dixon DL, Mayne GC, Griggs KM, De Pasquale CG, Bersten AD (2013) Chronic elevation of pulmonary microvascular pressure in chronic heart failure reduces bi-directional pulmonary fluid flux. Eur J Heart Fail 15(4):368–375 23248216 10.1093/eurjhf/hfs201

[60] Lichtenstein D, Mézière G, Biderman P, Gepner A, Barré O (1997) The comet-tail artifact. An ultrasound sign of alveolar-interstitial syndrome. Am J Respir Crit Care Med 156(5):1640–1646 9372688 10.1164/ajrccm.156.5.96-07096

[61] Copetti R, Soldati G, Copetti P (2008) Chest sonography: a useful tool to differentiate acute cardiogenic pulmonary edema from acute respiratory distress syndrome. Cardiovasc Ultrasound 6:16 18442425 10.1186/1476-7120-6-16 PMC2386861

[62] Gargani L, Volpicelli G (2014) How i do it: lung ultrasound. Cardiovasc Ultrasound 12:25 24993976 10.1186/1476-7120-12-25 PMC4098927

[63] Volpicelli G, Elbarbary M, Blaivas M, International Liaison Committee on Lung Ultrasound (ILC-LUS) for International Consensus Conference on Lung Ultrasound (ICC-LUS) et al (2012) International evidence-based recommendations for point-of-care lung ultrasound. Intensive Care Med 38(4):577–591 22392031 10.1007/s00134-012-2513-4

[64] Agricola E, Bove T, Oppizzi M et al (2005) “Ultrasound comet-tail images”: a marker of pulmonary edema: a comparative study with wedge pressure and extravascular lung water. Chest 127(5):1690–1695 15888847 10.1378/chest.127.5.1690

[65] Yu CJ, Yang PC, Wu HD, Chang DB, Kuo SH, Luh KT (1993) Ultrasound study in unilateral hemithorax opacification. Image comparison with computed tomography. Am Rev Respir Dis 147(2):430–434 8430970 10.1164/ajrccm/147.2.430

[66] Lichtenstein D, Goldstein I, Mourgeon E, Cluzel P, Grenier P, Rouby JJ (2004) Comparative diagnostic performances of auscultation, chest radiography, and lung ultrasonography in acute respiratory distress syndrome. Anesthesiology 100(1):9–15 14695718 10.1097/00000542-200401000-00006

[67] Blackmore CC, Black WC, Dallas RV, Crow HC (1996) Pleural fluid volume estimation: a chest radiograph prediction rule. Acad Radiol 3(2):103–109 8796649 10.1016/s1076-6332(05)80373-3

[68] Rahman NM, Singanayagam A, Davies HE et al (2010) Diagnostic accuracy, safety and utilisation of respiratory physician-delivered thoracic ultrasound. Thorax 65(5):449–453 20435870 10.1136/thx.2009.128496

[69] Chen HJ, Tu CY, Ling SJ et al (2008) Sonographic appearances in transudative pleural effusions: not always an anechoic pattern. Ultrasound Med Biol 34(3):362–369 17996356 10.1016/j.ultrasmedbio.2007.09.009

[70] Grimberg A, Shigueoka DC, Atallah AN, Ajzen S, Iared W (2010) Diagnostic accuracy of sonography for pleural effusion: systematic review. Sao Paulo Med J 128(2):90–95 20676576 10.1590/S1516-31802010000200009 PMC10938974

[71] Xirouchaki N, Magkanas E, Vaporidi K et al (2011) Lung ultrasound in critically ill patients: comparison with bedside chest radiography. Intensive Care Med 37(9):1488–1493 21809107 10.1007/s00134-011-2317-y

[72] Zhang H, Zhou Y, Cheng F et al (2023) Prognostic impact of lung ultrasound detected B-lines on hospitalised ischaemic heart failure with mildly reduced ejection fraction patients. Open Heart 10(2):e002480 38065587 10.1136/openhrt-2023-002480 PMC10711819

[73] Rivas-Lasarte M, Maestro A, Fernández-Martínez J et al (2020) Prevalence and prognostic impact of subclinical pulmonary congestion at discharge in patients with acute heart failure. ESC Heart Fail 7(5):2621–2628 32633473 10.1002/ehf2.12842 PMC7524099

[74] Palazzuoli A, Ruocco G, Beltrami M, Nuti R, Cleland JG (2018) Combined use of lung ultrasound, B-type natriuretic peptide, and echocardiography for outcome prediction in patients with acute HFrEF and HFpEF. Clin Res Cardiol 107(7):586–596 29532155 10.1007/s00392-018-1221-7

[75] Gargani L, Pang PS, Frassi F et al (2015) Persistent pulmonary congestion before discharge predicts rehospitalization in heart failure: a lung ultrasound study. Cardiovasc Ultrasound 13:40 26337295 10.1186/s12947-015-0033-4 PMC4558829

[76] Rivas-Lasarte M, Álvarez-García J, Fernández-Martínez J et al (2019) Lung ultrasound-guided treatment in ambulatory patients with heart failure: a randomized controlled clinical trial (LUS-HF study). Eur J Heart Fail 21(12):1605–1613 31667987 10.1002/ejhf.1604

[77] Öhman J, Harjola VP, Karjalainen P, Lassus J (2018) Focused echocardiography and lung ultrasound protocol for guiding treatment in acute heart failure. ESC Heart Fail 5(1):120–128 28960894 10.1002/ehf2.12208 PMC5793966

[78] Gustafsson M, Alehagen U, Johansson P (2015) Imaging congestion with a pocket ultrasound device: prognostic implications in patients with chronic heart failure. J Card Fail 21(7):548–554 25725475 10.1016/j.cardfail.2015.02.004

[79] Szabó IA, Gargani L, Morvai-Illés B et al (2022) Prognostic value of lung ultrasound in aortic stenosis. Front Physiol 13:838479 35480045 10.3389/fphys.2022.838479 PMC9037236

[80] Mancusi C, Bahlmann E, Basile C, Gerdts E (2022) New evidence about aortic valve stenosis and cardiovascular hemodynamics. High Blood Press Cardiovasc Prev 29(3):231–237 35438477 10.1007/s40292-022-00520-x PMC9050777

[81] Pellicori P, Shah P, Cuthbert J et al (2019) Prevalence, pattern and clinical relevance of ultrasound indices of congestion in outpatients with heart failure. Eur J Heart Fail 21(7):904–916 30666769 10.1002/ejhf.1383

[82] Vahanian A, Beyersdorf F, Praz F, *et al*; ESC/EACTS Scientific Document Group. 2021 ESC/EACTS Guidelines for the management of valvular heart disease. Eur Heart J 2022;43(7):561–632. Erratum in: Eur Heart J 2022;43(21):2022. 10.1093/eurheartj/ehab395 34453165

[83] Al Deeb M, Barbic S, Featherstone R, Dankoff J, Barbic D (2014) Point-of-care ultrasonography for the diagnosis of acute cardiogenic pulmonary edema in patients presenting with acute dyspnea: a systematic review and meta-analysis. Acad Emerg Med 21(8):843–852 25176151 10.1111/acem.12435

[84] Mancusi C, Basile C, Spaccarotella C et al (2024) Novel strategies in diagnosing heart failure with preserved ejection fraction: a comprehensive literature review. High Blood Press Cardiovasc Prev 31(2):127–140 38489152 10.1007/s40292-024-00629-1 PMC11043114

